# Supplementary material for: Quantitative constraint-based computational model of tumor-to-stroma coupling via lactate shuttle
Source: Sci Rep. 2015 Jul 7;5:11880. doi: 10.1038/srep11880 (PMC4493718; doi:10.1038/srep11880)
Supplement: Supplementary Information [file srep11880-s1.pdf]

# Quantitative constraint-based computational model of tumor-to-stroma coupling via lactate shuttle

Fabrizio Capuani[1,⋆], Daniele De Martino[1,2], Enzo Marinari[1,4,†], Andrea De Martino[1,2,3,†],

**1** Dipartimento di Fisica, Sapienza Università di Roma, Piazzale A. Moro 2, Rome (Italy)

**2** Center for Life Nano Science@Sapienza, Istituto Italiano di Tecnologia, Viale Regina Elena 291, Rome (Italy)

**3** Soft and Living Matter Laboratory, Istituto di Nanotecnologia (CNR/NANOTEC), Consiglio Nazionale delle Ricerche, Rome (Italy)

**4** INFN sezione di Roma 1, Piazzale A. Moro 2, Rome (Italy)

⋆ Correspondence to fabrizio.capuani@uniroma1.it

† *Authors contributed equally*

## Text S1. Human Catabolic Core Network: biosynthetic reactions

Besides the major central carbon metabolic pathways, i.e. Glycolysis, Pentose Phosphate Pathway, Citric Acid Cycle, and Oxidative Phosphorylation, our model includes most reactions of Glutamate metabolism as well as lumped reactions describing the biosynthesis of all non-essential amino acids (i.e. the amino acids that can be synthesized by human cells) and palmitate, a key precursor of other fatty acids.

Amino acid biosynthetic processes can be divided roughly in two classes. The synthesis of tyrosine and cysteine requires respectively the essential amino acids phenylalanine and methionine. All other amino acids can instead be synthetized from glutamine plus metabolites from the central carbon pathways. The processes leading to amino acid synthesis are of various types, from simple single transaminase reactions to a complex chain of enzymatic reactions. In the latter case, by means of mass balance arguments we can lump together individual reaction steps to obtain effective reactions whose net output corresponds to the desired amino acid(s). In all cases, our starting points were the reactions and pathways included in the reference metabolic network reconstruction Recon-1 [21]. In the `.xml` file provided as Supporting Material, we include the list of the corresponding Recon-1 reactions that constitute the full pathway. In the following, we briefly outline these effective reactions.

- Glutamate synthesis: from glutamine via glutaminase (GLUN):

$$\text{gln\_L} + \text{H}_2\text{O} \rightarrow \text{glu\_L} + \text{NH}_4 \quad (1)$$

- Alanine and aspartate synthesis: from glutamate through the transaminase reactions

$$\text{akg} + \text{ala\_L} \leftrightarrow \text{glu\_L} + \text{pyr} \quad (2)$$

$$\text{akg} + \text{asp\_L} \leftrightarrow \text{glu\_L} + \text{oaa} \quad (3)$$

(akg = $\alpha$-ketoglutarate ; pyr = pyruvate ; oaa = oxaloacetate)

- Proline synthesis: from glutamate via glutamate 5-kinase (GLU5K), glutamate-5-semialdehyde dehydrogenase (G5SD), L-glutamate 5-semialdehyde dehydratase (G5SAD) and pyrroline-5-carboxylate reductase (P5CR):

$$\text{glu\_L} + \text{ATP} + (2)\text{NADPH} + (2)\text{H}^+ \rightarrow \text{pro\_L} + \text{ADP} + \text{Pi} + \text{H}_2\text{O} + (2)\text{NADP} \quad (4)$$

- Serine synthesis: from glutamate via phosphoglycerate dehydrogenase (PGCD), phosphoserine transaminase (PSERT) and phosphoserine phosphatase (PSP):

$$\text{3pg} + \text{NAD} + \text{H}_2\text{O} + \text{glu\_L} \rightarrow \text{ser\_L} + \text{akg} + \text{NADH} + \text{Pi} + \text{H}^+ \quad (5)$$

(3pg = 3-phosphoglyceric acid)

- Glycine synthesis: from serinevia glycine hydroxymethyltransferase (GHMT), methylenetetrahydrofolate dehydrogenase (MTHFD), methenyltetrahydrofolate cyclohydrolase (MTHFC) and formyltetrahydrofolate dehydrogenase (FDH):

$$\text{ser\_L} + \text{H}_2\text{O} + (2)\text{NADP} \rightarrow \text{gly} + \text{CO}_2 + (2)\text{NADPH} + (2)\text{H}^+ \quad (6)$$

- Arginine synthesis: from glutamate and aspartate via glutamate 5-kinase (GLU5K), glutamate-5-semialdehyde dehydrogenase (G5SD), ornithine transaminase (ORNTA), carbamoyl-phosphate

synthase (CBPS), ornithine carbamoyltransferase (OCBT), argininosuccinate synthase (ARGSS) and argininosuccinate lyase (ARGSL):

$$\text{asp\_L} + \text{glu\_L} + \text{gln\_L} + (5)\text{ATP} + \text{NADPH} + (3)\text{H}_2\text{O} + \text{CO}_2 \rightarrow$$
$$\rightarrow \text{arg\_L} + \text{fum} + \text{akg} + (5)\text{ADP} + (5)\text{Pi} + \text{NADP} + (5)\text{H}^+ \quad (7)$$

- Asparagine synthesis: from aspartate and glutamine via asparagine synthase (ASNS):

$$\text{asp\_L} + (2)\text{ATP} + \text{gln\_L} + (2)\text{H}_2\text{O} \rightarrow (2)\text{ADP} + \text{asn\_L} + \text{glu} + (2)\text{H}^+ + (2)\text{Pi} \quad (8)$$

- Cysteine synthesis: from serine and methionine via methionine adenosyltransferase (METAT), adenosylhomocysteinase (AHC), cystathionine beta-synthase (CBS), cystathionine gamma-lyase (CSE), 2-Oxobutanoate dehydrogenase, Propionyl-CoA carboxylase (PCCA) and methylmalonyl-CoA mutase (MMM):

$$\text{met\_L} + \text{ser\_L} + \text{coa} + (4)\text{ATP} + \text{NAD} + (4)\text{H}_2\text{O} \rightarrow$$
$$\rightarrow \text{cys\_L} + \text{succoa} + (4)\text{ADP} + (4)\text{Pi} + (4)\text{H}^+ + \text{NADH} + \text{NH}_4 + CH_3 \quad (9)$$

(coa = coenzyme A ; succoa = succinyl-coa)

- Tyrosine synthesis: from phenylalanine via L-Phenylalanine hydroxylase, tetrahydrobiopterin oxidoreductase, tetrahydrobiopterin-4a-carbinolamine dehydratase and dihydropteridine reductase:

$$\text{phe\_L} + \text{NADH} + \text{O}_2 + \text{H}^+ \rightarrow \text{tyr\_L} + \text{NAD} + \text{H}_2\text{O} \quad (10)$$

- Palmitic acid synthesis:

$$(8)\text{accoa} + (23)\text{ATP} + (14)\text{NADPH} + (17)\text{H}_2\text{O} \rightarrow$$
$$\rightarrow \text{palmitate} + (8)\text{coa} + (14)\text{NADP} + (23)\text{ADP} + (23)\text{Pi} + (10)\text{H}^+ \quad (11)$$

(accoa = acetyl-coa)

In addition, we included the reactions for superoxyde reduction and $FADH_2$ oxidation, that are necessary to correctly account for the redox state of the cell. For sakes of simplicity, we lumped together the reactions catalyzed by superoxide dismutase, gluthatione peroxidase and glutathione reductase to the single reaction

$$\mathrm{NADPH} + \mathrm{O}_2^- + 2\mathrm{H}^+ \rightarrow \mathrm{NADP} + 2\mathrm{H}_2\mathrm{O} \quad . \tag{12}$$

Likewise, we unified the two reactions that oxidate $FADH_2$, thereby reducing ubiquinone by means of the Electron transport flavoprotein-ubiquinone oxidoreductase (ETF) to

$$\mathrm{FADH}_2 + \mathrm{Q10} \rightarrow \mathrm{FAD} + \mathrm{Q10H}_2 \ . \tag{13}$$

## Text S2. Maximizing the flux of precursors: results

In the main text, we show that lactate overflow and shuttle arises under both biomass and ATP maximization. Here, we show the metabolic flux patterns obtained when in the lactate donor the production of single biomass precursors are maximized, to assess the robustness of the lactate overflow and shuttle. To limit the amino acid productions, we have assumed a fixed maximum total influx of glutamine, phenylalanine and methionine (equals to 2 mmol/(gDWh)) and a variable overall glucose supply.

All objective functions we tested yield a lactate overflow with lactate shuttle towards the acceptor, suggesting that the crowding constraint is responsible for the effect. In particular, we recover high levels of lactate shuttling for palmitate optimization and moderate levels for the optimization of the production of amino acids. The only lactate shuttle truly related to an energetic imbalance is the one induced by palmitate optimization, while the others are similar in magnitude to the shuttling present in the absence of any optimization (i.e. for $\beta = 0$). In Fig. S2, we display the production flux and lactate shuttling profiles as a function of the total glucose supply for the two-cell system when the lactate donor maximizes palmitate, proline, cysteine, and serine, respectively. Other biosynthetic objective functions lead to similar features.

# Supporting Figures

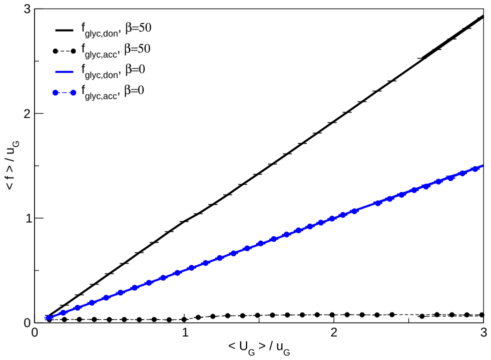


**(a)** Glucose intake

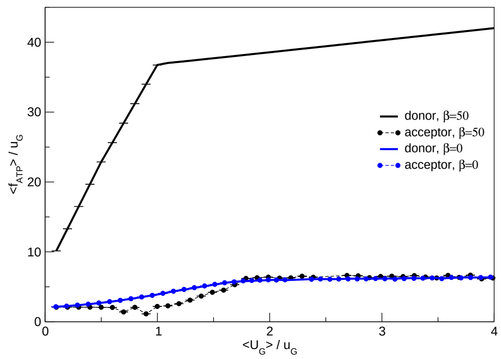


**(b)** ATP production

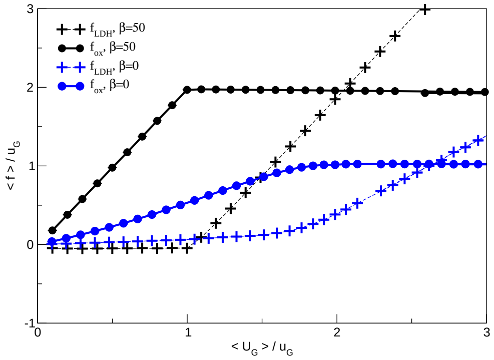


**(c)** Fluxes in donor cell

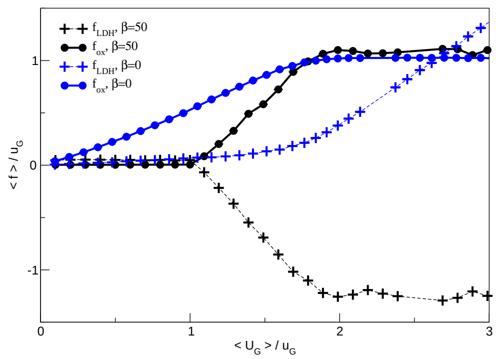


**(d)** Fluxes in acceptor cel

**Figure S1. Glucose intake, ATP production, and oxidative and fermentative fluxes in a donor-acceptor symmetric system.** (a) Glucose intakes for two coupled symmetric cells as a function of the total glucose available to the donor-acceptor pair. (b) ATP produced by the donor and the acceptor cells as a function of the total glucose available to the donor-acceptor pair. (c)–(d) Average flux through LDH (circles) and PDHm (crosses) as a function of the average glucose supplied to the two-cell system. Curves describe the behaviour obtained for two coupled symmetric HCCN cells with an ATP-maximizing donor (black lines, $\beta = 50$) or for an unbiased sampling of the two-cell solution space (blue lines, $\beta = 0$). Error bars, which represents s.e.m., are smaller than symbol sizes.

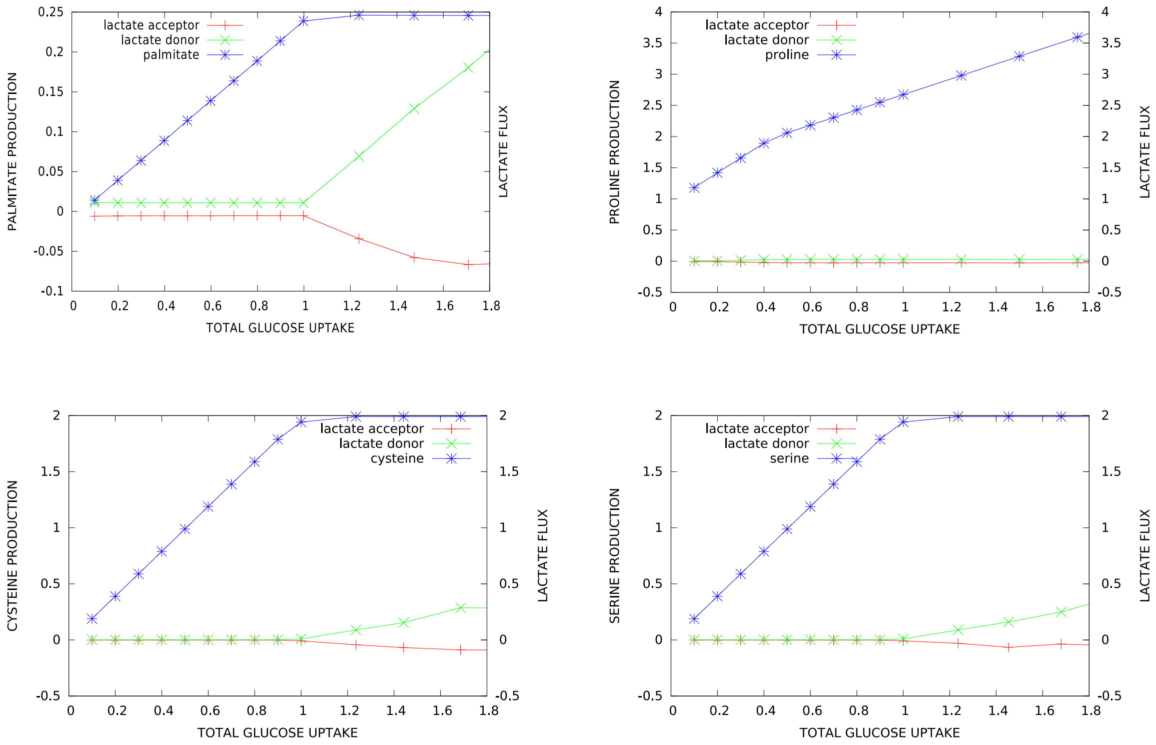


**Figure S2. Lactate shuttling for alternative objective function maximizations.** In blue we plot the flux that is maximized in the donor cell: palmitate (top left), proline (top right), cysteine (bottom left), and serine (bottom right) as a function of the total glucose supply, for a system formed by a lactate donor and a lactate acceptor. The lactate fluxes of donor and acceptor cells are depicted in green and red, respectively. A negative flux correspond to lactate influx.

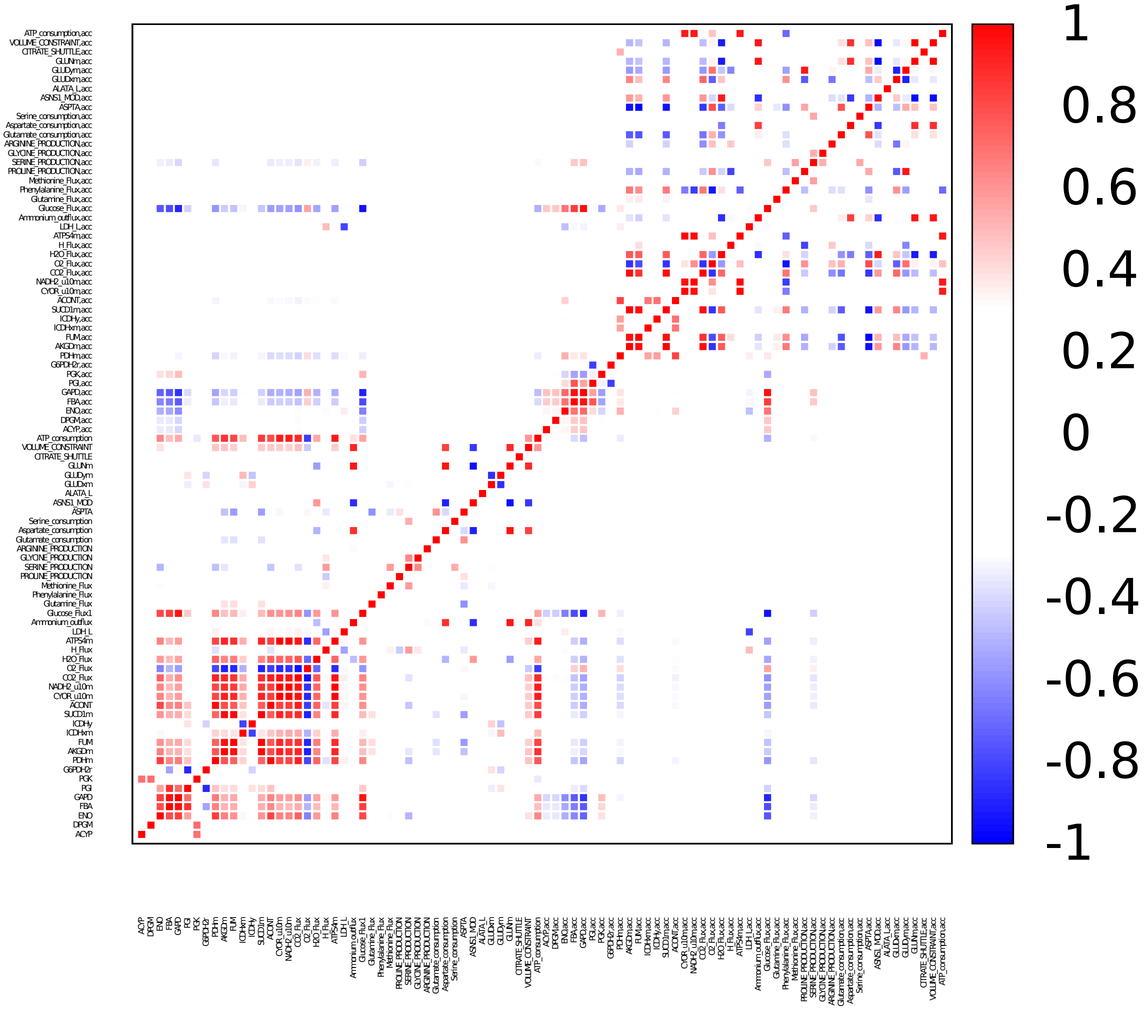


**Figure S3. Pearson correlation coefficients among all fluxes of lactate donor and lactate acceptor for maximum glucose uptake $U_G = 0.3$.** The fluxes of the acceptor cell are identified by the trailing letters "acc".

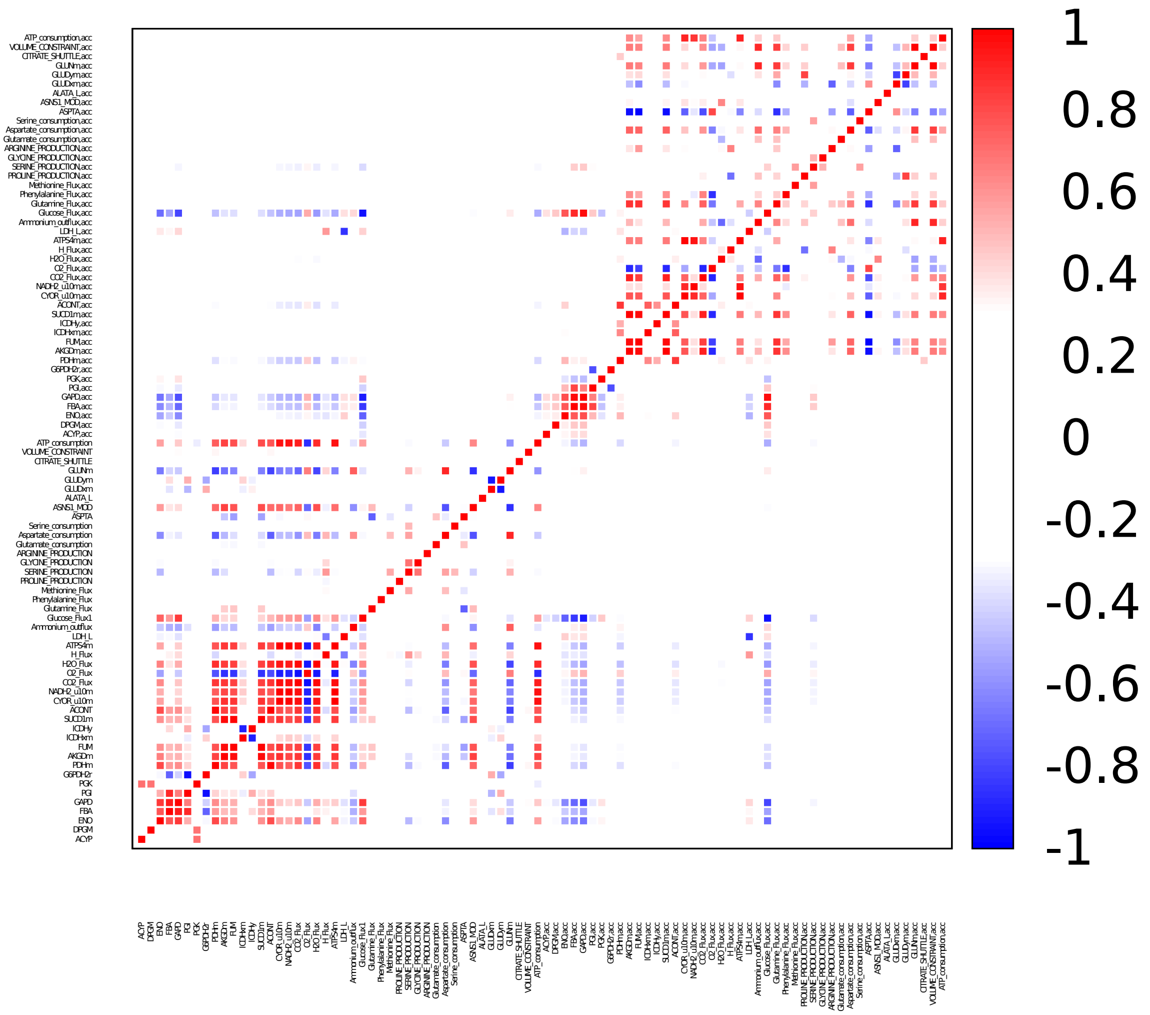


**Figure S4. Pearson correlation coefficients among all fluxes of lactate donor and lactate acceptor for maximum glucose uptake $U_G = 1$.** The fluxes of the acceptor cell are identified by the trailing letters “acc”.

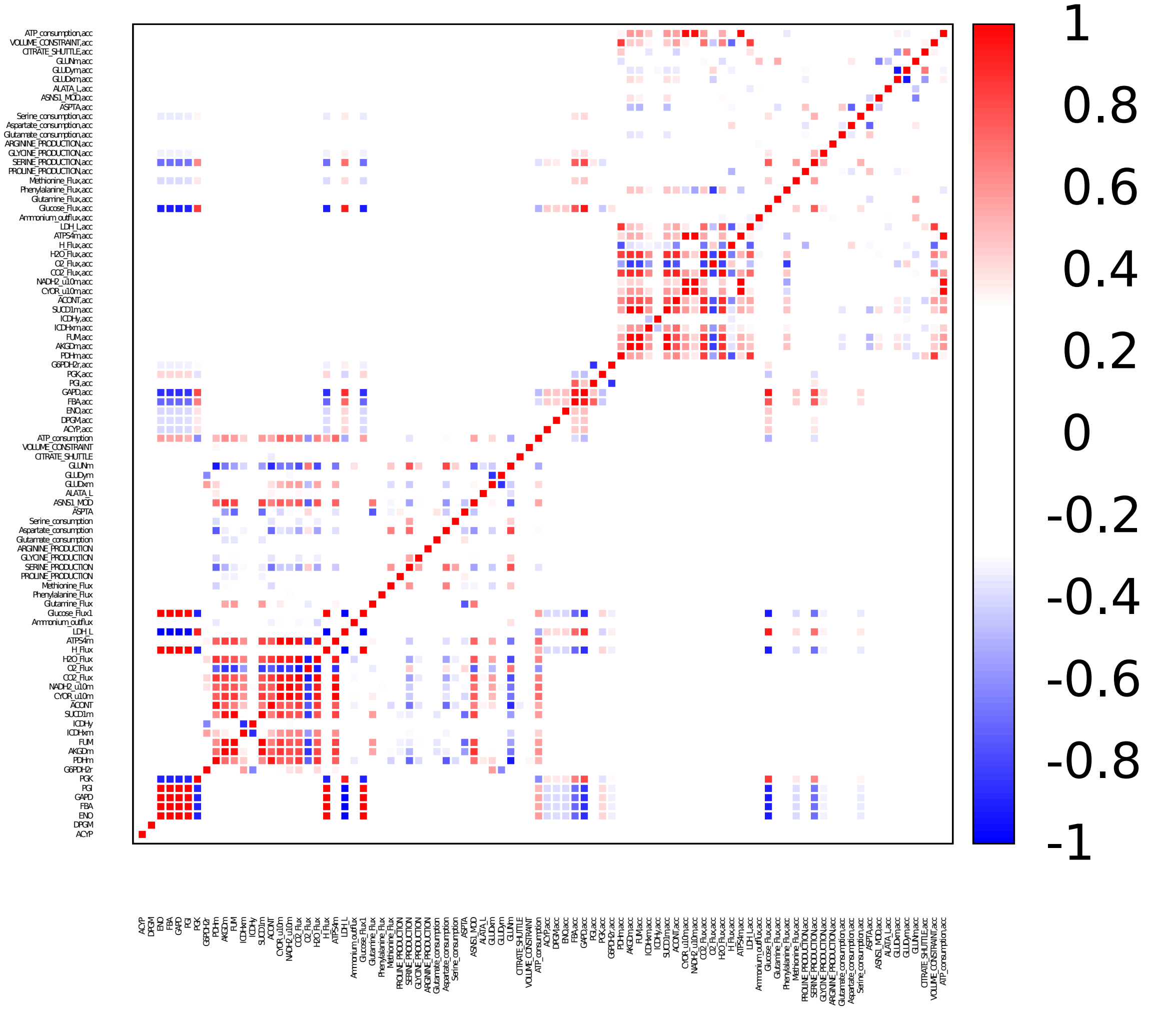


**Figure S5. Pearson correlation coefficients among all fluxes of lactate donor and lactate acceptor for maximum glucose uptake $U_G = 3$.** The fluxes of the acceptor cell are identified by the trailing letters "acc".

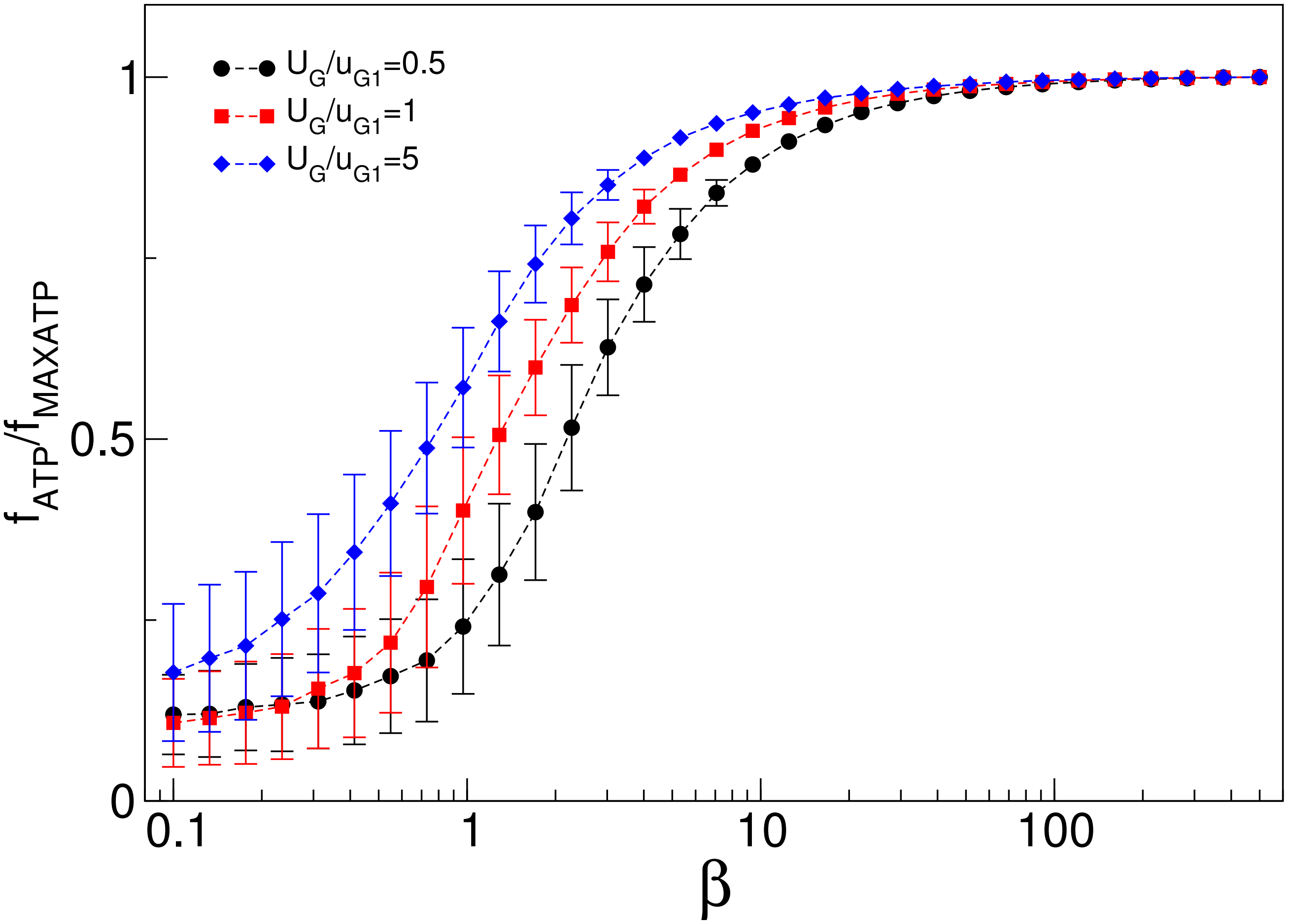


**Figure S6. By increasing $\beta$, an HCCN increases the ATP production eventually saturating the capacity at around $\beta \simeq 50$.** We display normalized ATP production fluxes for maximal glucose supply $U_{MAX}$ equals to 0.5 (black circles), 1 (red squares), and 5 (blu diamonds).

# Supporting Tables

| Variable symbol | Flux identified |
|---|---|
| $U_{\mathrm{G}}$ | Glucose supply |
| $f_{\mathrm{glyc}}$, $f_{\mathrm{HEX}}$ | Glycolysis flux |
| $f_{\mathrm{ox}}$, $f_{\mathrm{PDH}}$ | Oxidative phosphorylation flux |
| $f_{\mathrm{LDH}}$ | Flux through lactate dehydrogenase |
| $f_{\mathrm{ATP}}$ | ATP production |

**Table S1. List of more relevant variables appearing in the manuscript.**

| Metabolite short name | Metabolite extended name |
|---|---|
| 13 DPG | 3-Phospho-D-glyceroyl phosphate |
| 23 DPG | 2,3-Disphospho-D-glycerate |
| 2 PG | D-Glycerate 2-phosphate |
| 3 PG | 3-Phospho-D-glycerate |
| 6 PGC | 6-Phospho-D-gluconate |
| 6 PGL | 6-phospho-D-glucono-1,5-lactone |
| ACCoA | Acetyl-CoA |
| ADP | Adenosine diphosphate |
| AKG | 2-Oxoglutarate |
| ATP | Adenosine triphosphate |
| CIT | Citrate |
| $CO_2$ | Carbon dioxyde |
| CoA | Coenzyme A |
| DHAP | Dihydroxyacetone phosphate |
| E4P | D-Erythrose 4-phosphate |
| F6P | D-Fructose 6-phosphate |
| FAD | Flavin adenine dinucleotide oxidized |
| $FADH_2$ | Flavin adenine dinucleotide reduced |
| FDP | D-Fructose 1,6-bisphosphate |
| FICYTC | Ferricytochrome C |
| FOCYTC | Ferrocytochrome C |
| FUM | Fumarate |
| G3P | Glyceraldehyde 3-phosphate |
| G6P | D-Glucose 6-phosphate |
| GLC | D-Glucose |
| $H_2O$ | Water molecule |
| H[M] | Hydrogen ion as an electromotive force (mitochondrial) |
| H | Hydrogen ion in cytoplasm |
| ICIT | Isocitrate |
| LAC−L | L-Lactate |
| MAL−L | L-Malate |
| NAD | Nicotinamide adenine dinucleotide |
| NADH | Nicotinamide adenine dinucleotide - reduced |
| NADP | Nicotinamide adenine dinucleotide phosphate |
| NADPH | Nicotinamide adenine dinucleotide phosphate - reduced |
| $O_2$ | Molecular oxygen |
| $O_2S$ | Superoxide anion |
| OAA | Oxaloacetate |
| PEP | Phosphoenolpyruvate |
| Pi | Phosphate |
| PYR | Pyruvate |
| Q10 | Ubiquinone-10 |
| $Q10\,H_2$ | Ubiquinol-10 |
| R5P | alpha-D-Ribose 5-phosphate |
| RU5P−D | D-Ribulose 5-phosphate |
| S7P | Sedoheptulose 7-phosphate |
| SUCC | Succinate |
| SUCCoA | Succinyl-CoA |
| XU5P−D | D-Xylulose 5-phosphate |
| gln_L | Glutamine |
| glu_L | Glutamate |
| $NH_4$ | Ammonia |
| $CH_3$ | Methil group |
| asp_L | Aspartate |
| ala_L | Alanine |
| asn_L | Asparagine |
| pro_L | Proline |
| ser_L | Serine |
| gly | Glycine |
| arg_L | Arginine |
| cys_L | Cysteine |
| met_L | Methionine |
| tyr_L | Tyrosine |
| phe_L | Phenylalanine |
| hdca | Palmitic acid |

**Table S2. List of metabolites appearing in a single HCCN model.**

| Enzyme | Reaction |
|---|---|
| ACONT | $CIT \longleftrightarrow ICIT$ |
| ACYP | $13\,DPG + H_2O \longrightarrow 3\,PG + H + Pi$ |
| AKGDm | $AKG + CoA + NAD \longrightarrow CO_2 + NADH + SUCCoA$ |
| ATPS4 m | $ADP + 4H + Pi \longrightarrow ATP + 3H[M] + H_2O$ |
| CSm | $ACCoA + H_2O + OAA \longrightarrow CIT + CoA + H[M]$ |
| CYOOm3 | $4focytC + 7.92H[M] + O_2 \longrightarrow 4ficytC + 4H + 1.96H_2O + 0.02{O_2}^-$ |
| CYOR_u10m | $2ficytC + 2H[M] + Q_{10}H_2 \longrightarrow 2focytC + 4H + Q_{10}$ |
| DPGM | $13\,DPG \longleftrightarrow 23\,DPG + H$ |
| DPGase | $23\,DPG + H_2O \longrightarrow 3\,PG + Pi$ |
| ENO | $2\,PG \longleftrightarrow H_2O + PEP$ |
| FBA | $FDP \longleftrightarrow DHAP + G3P$ |
| FUM | $FUM + H_2O \longleftrightarrow MAL{-}L$ |
| G6PDH2r | $G6P + NADP \longleftrightarrow 6\,PGL + H + NADPH$ |
| GAPD | $G3P + NAD + Pi \longleftrightarrow 13\,DPG + H + NADH$ |
| GND | $6\,PGC + NADP \longrightarrow CO_2 + NADPH + RU5P{-}D$ |
| HEX1 | $ATP + GLC \longrightarrow ADP + G6P + H$ |
| ICDHxm | $ICIT + NAD \longrightarrow AKG + CO_2 + NADH$ |
| ICDHy | $ICIT + NADP \longrightarrow AKG + CO_2 + NADPH$ |
| LDH | $LAC{-}L + NAD \longleftrightarrow H + NADH + PYR$ |
| MDH | $MAL{-}L + NAD \longleftrightarrow H + NADH + OAA$ |
| NADH2_u10m | $5H + NADH + Q10 \longrightarrow 4H + NAD + Q10\,H_2$ |
| PDHm | $CoA + NAD + PYR \longrightarrow ACCoA + CO_2 + NADH$ |
| PFK | $ATP + F6P \longrightarrow ADP + FDP + H$ |
| PGI | $G6P \longleftrightarrow F6P$ |
| PGK | $3\,PG + ATP \longleftarrow 13\,DPG + ADP$ |
| PGL | $6\,PGL + H_2O \longrightarrow 6\,PGC + H$ |
| PGM | $2\,PG \longleftrightarrow 3\,PG$ |
| PYK | $ADP + H + PEP \longrightarrow ATP + PYR$ |
| RPE | $RU5P{-}D \longleftrightarrow XU5P{-}D$ |
| RPI | $R5P \longleftrightarrow RU5P{-}D$ |
| SUCD1m | $FAD + SUCC \longleftrightarrow FADH_2 + FUM$ |
| SUCOASm | $ATP + CoA + SUCC \longleftrightarrow ADP + Pi + SUCCoA$ |
| TALA | $G3P + S7P \longleftrightarrow E4P + F6P$ |
| TKT1 | $R5P + XU5P{-}D \longleftrightarrow G3P + S7P$ |
| TKT2 | $E4P + XU5P{-}D \longleftrightarrow F6P + G3P$ |
| TPI | $DHAP \longleftrightarrow G3P$ |
| O2S reduction | $NADPH + {O_2}^- + 2\,H \longrightarrow NADP + 2H_2O$ |
| FAD regeneration | $Q10 + FADH_2 \longrightarrow Q10H_2 + FAD$ |
| ATP consumption | $ATP + H_2O \longrightarrow ADP + Pi + H$ |

**Table S3. Internal reactions of an HCCN with the corresponding enzyme that catalyzes it: main catabolic pathways.**

| Enzyme | Reaction |
|---|---|
| ALATA_L | AKG + ala_L $\longleftrightarrow$ glu_L + PYR |
| ASPTA | AKG + asp_L $\longleftrightarrow$ glu_L + OAA |
| GLUDxm | glu_L + $H_2O$ + NAD $\longrightarrow$ AKG + H + NADH + $NH_4$ |
| GLUDym | glu_L + $H_2O$ + NADP $\longrightarrow$ AKG + H + NADPH + $NH_4$ |
| GLUN | gln_L + $H_2O$ $\longrightarrow$ glu_L + $NH_4$ |
| Tyrosine production | phe_L + NADH + $O_2$ + h $\longrightarrow$ tyr_L + NAD + $H_2O$ |
| Asparagine production | asp_L + (2 )ATP + gln_L + (2 )$H_2O$ $\longrightarrow$ (2 )ADP + asn_L + glu_L + (2 )H + (2 )Pi |
| Proline production | glu_L + ATP + (2 )NADPH + $2H^+$ $\longrightarrow$ pro_L + ADP + Pi + $H_2O$ + (2 )NADP |
| Serine production | 3 PG + NAD + $H_2O$ + glu_L $\longrightarrow$ ser_L + AKG + NADH + Pi + H |
| Glycine production | ser_L + $H_2O$ + (2 )NADP $\longrightarrow$ gly + $CO_2$ + (2 )NADPH + (2 )H |
| Arginine production | asp_L + glu_L + gln_L + (5 )ATP + NADPH + $3H_2O$ + $CO_2$ $\longrightarrow$ arg_L + FUM + AKG + (5 )ADP + (5 )Pi + 5H + NADP |
| Cysteine production | met_L + ser_L + COA + (4 )ATP + NAD + $CO_2$ + (4 )$H_2O$ $\longrightarrow$ cys_L + SUCCOA + (4 )ADP + (4 )Pi + 4H + NADH + $NH_4$ + $CO_2$ + $CH_3$ |
| Palmitic acid production | (8 )ACCOA + (23 )ATP + 8NADH + (6 )NADPH + $17H_2O$ $\longrightarrow$ hdca + (8 )COA + (8 )NAD + (6 )NADP + (23 )ADP + (23 )Pi + (10 )H |

**Table S4. Internal reactions of an HCCN with the corresponding enzyme that catalyzes it: glutamate metabolism and anabolic effective reactions.**

| Description | Lower bound | Upper bound | Reaction |
|---|---|---|---|
| Flux of carbon dioxide | -1000 | 1000 | $CO_2 \longleftrightarrow$ out |
| Flux of oxygen | -1000 | 1000 | $O_2 \longleftrightarrow$ out |
| Flux of water | -1000 | 1000 | $H_2O \longleftrightarrow$ out |
| Flux of ion hydrogen | -1000 | 1000 | H $\longleftrightarrow$ out |
| Glucose intake | 0 | $U_G$ | in $\longleftarrow$ GLC |
| Lactate flux | 0<br>-1000 | 1000<br>1000 | LAC-L $\longrightarrow$ out (donor)<br>LAC-L $\longleftrightarrow$ out (acceptor) |
| Glutamine intake | 0 | 1 | in $\longleftarrow$ gln_L |
| Phenylalanine intake | 0 | 1000 | in $\longleftarrow$ phe_L |
| Methionine intake | 0 | 1000 | in $\longleftarrow$ met_L |
| Ammonia flux | -1000 | 1000 | $NH_4 \leftrightarrow$ out |
| Methyl group flux | -1000 | 1000 | $CH_3 \leftrightarrow$ out |

**Table S5. Exchange reactions of an HCCN with the corresponding bounds.** In this model, we assume that carbon dioxide, water, oxygen, and proton can freely diffuse in and out of the cell. The maximum glucose intake for each cell cannot exceed the total glucose supply. Lactate flux distinguishes donor and acceptor cells as the donor can only secrete lactate, while the acceptor can also intake lactate. In Table S8, the constraints on glucose and lactate fluxes for the donor-acceptor couple are presented.

| Enzyme | Lower bound | Upper bound | Enzyme extended name |
|---|---|---|---|
| ACONT | -1000 | 1000 | Aconitase |
| ACYP | 0 | 1000 | Acylphosphatase |
| AKGDm | 0 | 1000 | 2-Oxoglutarate dehydrogenase |
| ATPS4m | 0 | 1000 | ATP synthase |
| CSm | 0 | 1000 | Citrate synthase |
| CYOOm3 | 0 | 1000 | Cytochrome C oxidase, mitochondrial Complex IV |
| CYOR_u10m | 0 | 1000 | Ubiquinol-6 cytochrome C reductase, Complex III |
| DPGM | -1000 | 1000 | Diphosphoglyceromutase |
| DPGase | 0 | 1000 | Diphosphoglycerate phosphatase |
| ENO | -1000 | 1000 | Enolase |
| FBA | -1000 | 1000 | Fructose-bisphosphate aldolase |
| FUM | -1000 | 1000 | Fumarase |
| G6PDH2r | -1000 | 1000 | Glucose 6-phosphate dehydrogenase |
| GAPD | -1000 | 1000 | Glyceraldehyde-3-phosphate dehydrogenase |
| GND | 0 | 1000 | Phosphogluconate dehydrogenase |
| HEX1 | 0 | 1000 | Hexokinase |
| ICDHxm | 0 | 1000 | Isocitrate dehydrogenase |
| ICDHy | 0 | 1000 | Isocitrate dehydrogenase |
| LDH | -1000 | 1000 | Lactate dehydrogenase |
| MDH | -1000 | 1000 | Malate dehydrogenase |
| NADH2_u10m | 0 | 1000 | NADH dehydrogenase, mitochondrial |
| PDHm | 0 | 1000 | Pyruvate dehydrogenase |
| PFK | 0 | 1000 | Phosphofructokinase |
| PGI | -1000 | 1000 | Glucose-6-phosphate isomerase |
| PGK | -1000 | 0 | Phosphoglycerate kinase |
| PGL | 0 | 1000 | 6-phosphogluconolactonase |
| PGM | -1000 | 1000 | Phosphoglycerate mutase |
| PYK | 0 | 1000 | Pyruvate kinase |
| RPE | -1000 | 1000 | Ribulose-5-phosphate 3-epimerase |
| RPI | -1000 | 1000 | Ribose-5-phosphate isomerase |
| SUCD1m | -1000 | 1000 | Succinate dehydrogenase |
| SUCOASm | -1000 | 1000 | Succinate–CoA ligase |
| TALA | -1000 | 1000 | Transaldolase |
| TKT1 | -1000 | 1000 | Transketolase |
| TKT2 | -1000 | 1000 | transketolase |
| TPI | -1000 | 1000 | Triose-phosphate isomerase |
| Lumped reaction | 0 | 1000 | Reduction of superoxyde anion |
| Lumped reaction | 0 | 1000 | FAD regeneration |
| Effective reaction | $f_{\mathrm{ATP}}^{\mathrm{min}}$ | 1000 | ATP hydrolysis |

**Table S6. Internal reactions of an HCCN with the corresponding bounds: catabolic pathways.**

| Enzyme | Lower bound | Upper bound | Enzyme extended name |
|---|---|---|---|
| ALATA_L | -1000 | 1000 | alanine transaminase |
| ASPTA | -1000 | 1000 | aspartate transaminase |
| GLUDxm | -1000 | 1000 | glutamate dehydrogenase (NAD) |
| GLUDym | 0 | 1000 | glutamate dehydrogenase (NADP) |
| GLUN | 0 | 1000 | glutaminase |
| Lumped reaction | 0 | 1000 | Tyrosine production |
| Lumped reaction | 0 | 1000 | Asparagine production |
| Lumped reaction | 0 | 1000 | Proline production |
| Lumped reaction | 0 | 1000 | Serine production |
| Lumped reaction | 0 | 1000 | Glycine production |
| Lumped reaction | 0 | 1000 | Arginine production |
| Lumped reaction | 0 | 1000 | Cysteine production |
| Lumped reaction | 0 | 1000 | Palmitic acid production |

**Table S7. Internal reactions of an HCCN with the corresponding bounds: glutamate metabolism and anabolic effective reactions.**

| Variable constrained | Lower bound | Upper bound | Variable expressed by reactions |
|---|---|---|---|
| Donor resource | 0 | $\Phi_{\mathrm{ATP}}$ | $a_{\mathrm{glyc}} f_{\mathrm{HEX,don}} + a_{\mathrm{ox}} f_{\mathrm{PDH,don}} + a_{\mathrm{LDH}} f_{\mathrm{LDH,don}}$ |
| Acceptor resource | 0 | $\Phi_{\mathrm{ATP}}$ | $a_{\mathrm{glyc}} f_{\mathrm{HEX,acc}} + a_{\mathrm{ox}} f_{\mathrm{PDH,acc}} + a_{\mathrm{LDH}} \lvert f_{\mathrm{LDH,acc}} \rvert$ |
| Total glucose intake | 0 | $U_{\mathrm{G}}$ | $U_{\mathrm{G,don}} + U_{\mathrm{G,acc}}$ |
| Total lactate flux | 0 | 1000 | $U_{\mathrm{LAC,don}} + U_{\mathrm{LAC,acc}}$ |

**Table S8. Constraints on maximum resources available for ATP production and on total glucose and lactate fluxes.** The first column contains a description of the variable that is constrained, while the last column the variable expressed as a function of the elementary fluxes of the metabolic network. The second and the third column contain the minimum and the maximum value that the variable can take, respectively. $\Phi_{\mathrm{ATP}}$ is set to 0.4 and states that at most 40% of the cellular resources can be devoted to ATP production. The sum of the glucose intaken by donor and acceptor cannot exceed the total glucose supply $U_{\mathrm{G}}$. The constraint on total lactate flux establishes that there is no external source of lactate in the system and that the donor-acceptor couple can only produce lactate.

| Metabolite | Biomass coefficient |
|---|---|
| $H_2O$ | -20.651 |
| ATP | -20.651 |
| ADP | 20.651 |
| H | 20.651 |
| Pi | 20.971 |
| glu_L | -0.38587 |
| asp_L | -0.35261 |
| asn_L | -0.27942 |
| ala_L | -0.50563 |
| cys_L | -0.046571 |
| gln_L | -0.326 |
| gly | -0.53889 |
| ser_L | -0.39253 |
| arg_L | -0.35926 |
| met_L | -0.15302 |
| tyr_L | -0.15967 |
| phe_L | -0.25947 |
| pro_L | -0.41248 |
| hdca | -0.112 |
| R5P | -0.045 |
| G6P | -0.275 |

**Table S9. Biomass objective function coefficients adapted from [47].**
